# Gun Violence and Psychopathy Among Female Offenders

**DOI:** 10.3389/fpsyg.2022.873305

**Published:** 2022-06-09

**Authors:** Nicholas D. Thomson

**Affiliations:** Virginia Commonwealth University, Richmond, VA, United States

**Keywords:** psychopathy, violence, gun violence, firearm-related crime, female offender

## Abstract

Research exploring risk factors of gun violence is limited, especially research involving women as perpetrators of violence. Yet, women account for 18–21% of convicted violent crime. The present study aimed to test if psychopathy, a notable risk factor for violence, was related to past convictions of gun violence, general forms of violence, and non-violent crime. In a sample of 206 female offenders, multinomial logistic regressions assessed how interpersonal, affective, and behavioral psychopathic traits increased the likelihood of women belonging to the gun violence group, a violent crime group, and a non-violent crime group. Results showed the interpersonal and affective facets increased the likelihood of women belonging to the gun violence group compared to both the violent and non-violent crime groups. The behavioral facet increased the likelihood of women belonging to the violent crime group when compared to the gun violence and non-violent crime groups. These results suggest that gun violence has different risk factors than violent and non-violent crime. This line of inquiry indicates that existing violence prevention strategies may need to be modified to address gun violence.

## Introduction

The firearm violence epidemic is a major public health problem. It is estimated that every day in the US, approximately 100 people fall casualty to gun violence (Evans and Anthony, 2018) and in 2019 over 14,000 people died from firearm homicide (CDC, 2019). Firearm homicides account for ∼75% of all homicides in the US (CDC, 2019). Compounding the tragic loss of life is the enormous economic burden of gun violence, which totals an estimated $229 billion annually in direct and indirect costs (U.S. Congress Joint Economic Committee and Democratic Staff, 2019). Although gun violence has reached an epidemic scale, there has been a lack of research on this topic due to federal funding restrictions on gun violence research for over 20 years. This has left gun violence prevention scientists with a dearth of empirical evidence on how to best intervene, impeding the development of evidence-informed gun violence interventions. Nevertheless, it is possible that gun violence shares similar risk factors as general forms of violence, and similar protective factors that buffer against general forms of violence may also buffer against gun violence. If this is found to be true, then existing violence interventions could be effective for cross-cutting violence prevention, including gun violence. Currently, there is not enough empirical evidence to support this assertion, at least from the psychological sciences. Thus, to prevent gun violence there is a critical need to understand the risk factors for gun violence, and if these risk factors are different from general forms of violence. If the risk factors are different for gun violence, then current violence intervention programs need to be tailored to increase their efficacy. One of the most widely researched individual-level risk factors is psychopathy. Psychopathy is regarded as a robust predictor for future violence perpetration across population types (e.g., forensic, community, inpatient) for both men and women (Thomson et al., 2022). The goal of this study is to understand if psychopathy is related to gun violence, and if this association is different/similar from general violent crime (non-firearm related) in a female offender sample.

### Psychopathy and Gun Violence

Psychopathy is a personality disorder whereby biological, psychological, and social factors contribute to its development (Thomson, 2019). Psychopathy is considered one of the most important clinical constructs for criminal behavior (Hare, 1996; Hemphill et al., 1998) in men and women (Baskin-Sommers et al., 2013; Gray and Snowden, 2016; Reidy et al., 2016; Thomson, 2018). People high on psychopathic traits are responsible for committing over half of all violent crimes (Hare, 1993) and early research has found that psychopathic individuals were more likely to use a firearm in their violent crime when compared to non-psychopathic offenders (Hare and McPherson, 1984). The association between psychopathy and dangerous/violent behavior is why psychopathy is estimated to cost society over $460 billion annually, placing it as one of the most costliest psychiatric disorders (Kiehl and Hoffman, 2011). Although early conceptions of psychopathy and diagnostic measures of psychopathy use total psychopathy scores and cut-off scores, current research suggests that the dimensionality of psychopathy is advantageous for understanding correlates of psychopathy (Thomson, 2019) and female offender profiles (Carabellese et al., 2020a,b). Psychopathy, as measured by the Levenson Self-Report Scale (LSRP; Levenson et al., 1995), consists of 3-facets: the interpersonal, affective, and behavioral facets. The interpersonal facet of psychopathy includes symptoms of grandiosity, conning and manipulative behaviors, superficial charm, social dominance, and selfishness (Sellbom, 2011). The affective facet is characterized by callousness, lack of empathy, and a lack of remorse (Sellbom, 2011). The behavioral facet measures boredom susceptibility and unstable, angry, impulsive attitudes and behaviors (Garofalo et al., 2019; Thomson et al., 2020a). Research has demonstrated that the interpersonal facet is related to low agreeableness and higher levels of narcissism and moral disengagement (Garofalo et al., 2019). The affective facet is related to a lack of empathy, guilt, and morality, low agreeableness and conscientiousness, and higher levels of cold-heartedness (Garofalo et al., 2019). The behavioral facet is related to chronic antisocial behavior, emotion dysregulation, and impulsivity (Christian and Sellbom, 2016).

Recent research using total psychopathy scores has found no relation between psychopathy and gun violence in a youth sample (Gonzales and McNiel, 2019), while some research has found a positive association between total psychopathy scores and firearm carrying behaviors (Saukkonen et al., 2016). Whereas research involving male youths found that callous-unemotional (CU) traits, a similar construct to the affective facet in adults, was related to increased gun carrying and gun violence (Robertson et al., 2020). This may suggest that the affective features of psychopathy are related to greater risk of gun violence, however, this is yet to be tested in women. Although there have been no studies to date testing the psychopathy facet level associations with gun violence, research involving both men and women has demonstrated that the facet structure of psychopathy is beneficial for understanding sex differences and similarities in psychopathic traits and violence (Thomson et al., 2019b). Therefore, prior inconsistencies found in studies using total psychopathy scores (e.g., Saukkonen et al., 2016; Gonzales and McNiel, 2019) could be related to the divergent association between psychopathy facets and gun violence.

### Psychopathy and Violence in Women

In 2019, 18–21% of all violent crimes were committed by women (based on violent crime data where the offender’s sex was known (OJJDP, 2020; FBI, 2021). Between 2014 and 2019, women perpetrating violent crimes increased by ∼4.5% (OJJDP, 2020). With over 90,000 violent crimes committed by women in 2019, there is a clear need to understand the correlates of female violence, and more specifically to understand the prevalence and correlates of gun violence. This is especially important as firearms are involved in three-quarters of all homicides (CDC, 2019). Psychopathy prospectively predicts violence in women and men (Brown et al., 2015; Olver and Wong, 2015), and research looking at sex differences has highlighted important distinctions in how psychopathy facets relate to violence (Thomson et al., 2019a, 2020b). The interpersonal facet has been related to instrumental violence in forensic male samples (Walsh et al., 2009; Declercq et al., 2012), and a meta-analysis by Blais et al. (2014) found that men and women did not differ in this association. Research involving both men and women indicate that the behavioral facet is a consistent predictor and correlate of violent behavior (Douglas et al., 2005; Kennealy et al., 2010; Poythress et al., 2010; Chakhssi et al., 2014). By contrast, the affective facet in men is found to be unrelated or marginally related to violence (Hall et al., 2004; Edens et al., 2008; Walters and Heilbrun, 2010), whereas in women the affective facet is related to future prison violence (Thomson et al., 2016), physical aggression (Thomson et al., 2019b), violent crime (Thomson, 2017), and interpersonal violence and proactive aggression in the community (Thomson et al., 2018). Therefore, it seems that the affective facet may be a female-specific risk factor for violence, while the behavioral and interpersonal facets are more gender-neutral. This may highlight that violence interventions developed for women may need to target symptoms of affective psychopathic traits, or target moderators/mediators that link affective psychopathic traits to violence (e.g., physical abuse; Thomson et al., 2019a). However, it remains unknown if this association is similar for women who commit gun violence, and if psychopathy facets are differentially related to gun violence when compared to general forms of violence (e.g., non-firearm related violence). Because prior research has suggested that the affective facet in women is related to more severe and chronic forms of violence (Thomson et al., 2016), it may be that the affective facet is also related to gun violence, whereas the interpersonal and behavioral facets may be related to a wider variety of violence.

### The Present Study

The goal of the present study was to test if psychopathic traits increased the likelihood of female offenders having a past violent conviction. The aim of the present study was to test whether women convicted of a firearm-related violent crime differed on psychopathy facets when compared to women convicted of a violent crime (without a gun), and women convicted of non-violent crimes. Because weapon use during violence has been linked to instrumental forms of aggression (see Raine et al., 2006; RPQ item 21), and instrumental violence is linked to the interpersonal facet of psychopathy (Blais et al., 2014), it is expected that higher interpersonal facet scores will increase the likelihood of women belonging to the gun violence crime group and the violent crime group when compared to the non-violent group. Further, because research in women has found the affective facet related to more serious forms of violence (Thomson, 2017; Thomson et al., 2019b), it is expected that higher affective facet scores will increase the likelihood of women belonging to the gun violence crime group when compared to the violent crime and non-violent crime groups. Lastly, research has found the behavioral facet to be related to violence in general, it is expected that the behavioral facet would increase the likelihood of women belonging to the violent crime group when compared to the non-violent crime group.

## Materials and Methods

### Participants

For the present study, we recruited female prisoners to assess if psychopathic traits were related to gun violence, violence, or non-violent crime. Participants (*N* = 206, *M*_age_ = 37 years, age range: 20–61 years) were recruited from a women’s correctional facility that houses maximum, medium, and minimum custody level female offenders. Participants self-identified as Pacific Islander/Native Hawaiian (53%), Caucasian (27%), Asian-American (8%), and other ethnicities (12%; Native American, Native Alaskan, African American, Hispanic American, and Mexican). Sixty-seven percent of the sample completed a 12th-grade education or higher. The level of education was coded based on the number of schooling years completed. Thirty-five percent of the participants had been convicted of a violent crime, which included assault (33%), robbery (18%), threatening (18%), homicide (9%), manslaughter (7%), kidnapping (7%), attempted manslaughter (4%) negligent homicide (2%), and sexual assault (2%).

All eligible participants at the correctional facility were invited to participate in the study. Women incarcerated who were receiving intensive psychiatric treatment (*n* = 15) in the mental health unit or under suicide watch in the medical facility were not targeted for recruitment (*n* = 6). Participants were notified about the study during brief presentations in the cafeterias during lunchtime. Small groups of participants (*n* = ∼30) completed the paper surveys at desks in a classroom. The classroom environment provided privacy when completing the paper surveys. Participants did not receive any compensation or incentive for participation. Participants were informed prior to consent that their involvement was for research and not part of the correctional institutional files. The present study was approved by the institutional review board at the University of Hawai’i.

### Measures

#### Psychopathy

Participants completed the Levenson Self-Report Psychopathy (LSRP; Levenson et al., 1995) scale to measure the 3-facet model of psychopathy. The LSRP is a widely used measure of psychopathic traits and has been validated for use in female offender populations (see Brinkley et al., 2001; Thomson et al., 2016). The LSRP consists of 26 items reported in a Likert-type self-report format. Ratings range from 1 (disagree strongly) to 4 (agree strongly). The interpersonal facet [10 items (i.e., “In today’s world, I feel justified in doing anything I can get away with to succeed”)], affective facet [four reversed items (i.e., “I make a point of trying not to hurt others in pursuit of my goals”)], and antisocial facets (six items [i.e., “I have been in a lot of shouting matches with other people”]), factors showed low to adequate internal consistency (Cronbach’s α = 0.80, 0.68, and 0.87, respectively).

#### Violent Criminal History

Data were collected from official reports of the most recent criminal conviction(s) that the inmate was serving a prison sentence for. These data were available from prison file records. Consistent with Thomson (2020), violent crimes included homicide, attempted homicide, assault, sexual assault, weapons possession, robbery, and kidnapping. Three groups were created based on the evidence of a gun used in the violent crime (gun violence crime group), a violent crime without a gun (violent crime group), and a non-violent crime group. This resulted in 10% in the gun violence crime group (*n* = 21), 25% in violent crime group (*n* = 52), and 65% in the non-violent group (*n* = 133). Thus, in the present sample, gun violence has a prevalence of 29% among convicted violent offender women. The gun violent crime group mean scores on the LSRP facets were: interpersonal facet = 23.71 (*SD* = 8.58); affective facet = 10.10 (*SD* = 3.22); behavioral facet = 13.05 (*SD* = 3.14). The violent crime group mean scores on the LSRP facets were: interpersonal facet = 18.35 (*SD* = 6.38); affective facet = 7.33 (*SD* = 3.11); behavioral facet = 12.67 (*SD* = 4.52). The non-violent crime group mean scores on the LSRP facets were: interpersonal facet = 17.56 (*SD* = 4.83); affective facet = 7.25 (*SD* = 2.55); behavioral facet = 10.89 (*SD* = 3.17).

#### Data Analytic Plan

Statistical analyses were conducted using R Studio (R Core Team, 2016). To examine whether psychopathic traits (interpersonal, affective, and behavioral) increased the likelihood of being in the gun violence group, violent group, or non-violent group (categorical, group-defining variable), a series of multinomial logistic regressions were conducted using nnet package (Venables and Ripley, 2002). Odds ratios and their corresponding 95% confidence intervals were included to provide an index of effect sizes, with intervals furthest away from 1 indicating stronger effects. Analyses included age and education grade level as covariates.

## Results

### Psychopathy and Gun Violence

The multinomial logistic regression testing the association between psychopathy facets and the type of crime (e.g., gun violence, violent crime (without a firearm), and non-violent crime) was significant [*x*^2^(10, *N* = 206) = 48.42, *p* < 0.001]. Table 1 displays the result of the multinomial logistic regression. Higher interpersonal (OR = 1.14, *p* = 0.012) and affective facets (OR = 1.33, *p* = 0.003) and younger age (OR = 0.93, *p* = 0.033) increased the odds of being in the gun violence crime group when compared to the non-violent crime group and violent crime group (OR = 1.16, *p* = 0.009; OR = 1.34, *p* = 0.004; OR = 0.92, *p* = 0.025; respectively). The behavioral facet increased the odds of being in the violent group when compared to the non-violent group and gun violence group (OR = 1.16, *p* = 0.011; OR = 1.30, *p* = 0.023; respectively). Lower level of education increased the likelihood of women belonging to the violent group when compared to the non-violent crime group (OR = 0.77, *p* = 0.029) but not compared to the gun violence group (*p* = 0.808). Thus, younger age, and interpersonal and affective facets increased the likelihood of women belonging to the gun violence crime group, while the behavioral facet and lower education increased the likelihood of women belonging to the violent crime group.

**TABLE 1:** Criminal group comparisons on the 3-facet construct of psychopathy based on odds ratios (95% CI).

	Gun violent crime vs. ^a^Non-violent Crime	Violent crime vs. ^a^Non-violent crime	Gun violent crime vs. ^a^Violent crime
Age	0.93* (0.87–0.99)	1.01 (0.97–1.04)	0.92* (0.86–99)
Education	0.73 (0.50–1.11)	0.77* (0.61–97)	0.95 (0.62–1.45)
Interpersonal	1.14* (1.03–1.27)	0.98 (0.92–1.06)	1.16** (1.04–1.30)
Affective	1.33** (1.10–1.60)	0.99 (0.87–1.12)	1.34** (1.10–1.64)
Behavioral	0.90 (0.72–1.10)	1.16* (1.04–1.30)	0.77* (0.61–0.96)

^a^Reference group; *p < 0.05, **p < 0.01.

## Discussion

Psychopathy is a well-established risk factor for violence in both men and women (Thomson, 2021), however, research exploring the association between psychopathy and gun violence in adults is limited (e.g., Hare and McPherson, 1984). The present study has extended this early research by finding that psychopathy is associated with gun violence, and the facet level of psychopathy can distinguish women who perpetrated gun violence from women who committed general forms of violent crime and non-violent crime. This has important implications. This result underlines that gun violence in women has distinct risk factors when compared to violent and non-violent crime. Thus, existing violence interventions may need to tailor programming to address gun violence risk factors. However, given that this is one study, and a study that focuses only on incarcerated women and psychopathy, much more research is needed to unpack gun violence risk factors. Thus, I encourage future research to tease apart associations of gun violence from general forms of violence to help direct violence prevention scientists in the development of evidence-informed strategies to prevent gun violence.

The present study found the affective and interpersonal facets independently increased the likelihood of women belonging to the gun-related violent crime group when compared to the violent crime and non-violent crime groups. Although this is a novel finding it is consistent with research including women, whereby the affective facet is related to more severe forms of violence in women (Thomson et al., 2019b). Indeed, recent research has found that women who committed murder had higher interpersonal-affective scores when compared to men who had committed murder (Carabellese et al., 2020a). Given that gun violence is responsible for 75% of all homicides, it is logical that gun violence is more severe than violence more generally. Therefore, this result may be indicative of severity. To tease this association apart future research is encouraged to explore mediators that may explain the link between the affective facet and gun violence. Also related with past research in men and women, is that the interpersonal facet is related to instrumental forms of violence. Given that weapon carrying and weapon use could be considered as planned, especially during a crime, it is possible that the proactive nature of gun violence explains the relation with the interpersonal facet of psychopathy. Indeed, weapon carrying is included in measures of proactive aggression (Raine et al., 2006). Furthermore, interpersonal and affective psychopathic traits are related to social dominance (Roy et al., 2021), which is often displayed by robbers who use firearms to commit crimes (Mosselman et al., 2018). Researchers have also suggested that firearm carrying among youth with conduct problems is used to establish dominance among peers (Beardslee et al., 2018). Collectively, it may be that women with higher interpersonal facet scores use a firearm in their violence to increase dominance. Again, the specific mechanisms need to be tested in future research. Nevertheless, women who display the prototypical psychopathic traits, characterized by callousness, lack of empathy and remorse, superficially charming, conning and manipulative, and grandiose are more likely to have engaged in gun violence.

The behavioral facet was not positively related to gun violence but did increase the likelihood of women belonging to the general violent crime group when compared to the gun violence group and non-violent crime group. Because the present study parsed women who committed gun violence from those who were violent without a firearm, it may be that those remaining in the violent crime group perpetrated acts of violence with less planning, comparatively. Although this is speculative and beyond the scope of the present study design, past research has found the behavioral facet is related to reactive aggression in men (Wang et al., 2018) and women (Thomson et al., 2018). An alternative explanation beyond gun violence vs. general forms of violence being indicative of proactive and reactive violence, respectively, is simply that women who have higher levels of prototypical psychopathic traits that are characterized as “cold-blooded” perpetrators are more likely to use a firearm in their violent crime (Thomson, 2019). In contrast, women who scored high on the behavioral facet are characteristically impulsive, have poor emotion regulation, and get into conflict with others more often (e.g., LSRP item 6; gets into a lot of shouting matches). This conglomeration of personality and behavioral traits seems to place women at greater risk of violence across different contexts, while the interpersonal and affective facets seem to be context and/or severity specific (Thomson et al., 2019).

These results may have clinical implications. Women who committed gun violence were characteristically different from women who engaged in violent and non-violent crime. These women were more callous, lacked empathy and remorse, and were manipulative, superficially charming, egocentric, and dominant. In contrast, violent women (non-firearm related) were more impulsive and reported having poor emotion regulation. These types of violent offenders would require very different treatment approaches, for the health of the individual and other incarcerated women. For example, trauma-informed emotion regulation group therapies are often used in forensic settings to reduce violence and antisocial behavior (e.g., Dialectical Behavior Therapy, Linehan, 2015; Beyond Violence, Covington, 2013). These strategies may work for reducing impulsivity and anger for women high on behavioral psychopathic traits, which could reduce psychopathy-related violence.

In contrast, trauma-informed emotion regulation group therapies may not be ideal for women who have perpetrated gun violence and actually be detrimental to other women in the group. Offenders higher on interpersonal-affective psychopathy engage in a range of treatment interfering behaviors, such as “manipulative and coercive behaviors, being disruptive, attention seeking, antagonizing patients and staff” (Thomson, 2019, p. 148), and have greater rates of treatment drop out (Sewall and Olver, 2018; Thomson, 2019). This can impact the treatment for other women and staff burnout. Further, offenders with higher interpersonal-affective psychopathy may use group therapy sessions to understand other women’s vulnerabilities, which could be used for coercive and targeted manipulation (Thomson, 2019). Therefore, practitioner care should be taken to avoid placing women at risk of further victimization. Yet, women who engage in gun violence are a high need treatment group but may require specialist intervention. Instead, a more effective treatment for offenders with high interpersonal-affective traits to reduce the risk of gun violence may be to use group therapy for cognitive behavioral skills while avoiding vulnerable topics for other inmates (e.g., trauma), and to use an individual treatment approach for vulnerable topics and to increase overall retention by improving strong therapeutic alliance. This multi-pronged and intensive approach has received encouraging support for high-risk psychopathic male offenders (Sewall and Olver, 2018). In line with the Sewall and Olver (2018) statement on sexual offenders, it is proposed here that a goal of treatment for women who perpetrate gun violence who are higher on interpersonal and affective traits should be less about changing resistant psychopathic personality traits, but to break the link between interpersonal and affective traits and gun violence. In women, research has suggested that exposure to physical abuse plays a role in the link between the affective facet and violence (Thomson et al., 2019a), this may support the notion that individual sessions focused on strong therapeutic alliance using trauma-informed approaches may be beneficial for reducing gun violence among women. While this paper has explored potential treatment implications, it is clear that much more research is needed to replicate the present study findings and to understand the specific mechanisms behind psychopathy-related firearm violence.

The present study has several limitations that should be considered when interpreting the results. Although this is a large study of female offenders, it is not possible to generalize the results to male samples, nor is it possible to make sex-specific comparisons, especially as there is no existing research on psychopathy facets and firearm-related violent crime in men. The present study used a self-report measure of psychopathy. While self-report psychopathy is a widely accepted approach to measuring psychopathic traits in women, it would be important to replicate using other semi-structured clinical assessments (i.e., Psychopathy Checklist-Revised; Hare, 2003) and other self-reports (i.e., Psychopathic Personality Inventory-Revised; Lilienfeld and Widows, 2005). Next, as a measure of criminal behavior, past criminal records were coded. It is likely that some offenders used a firearm in violent crimes but this was not documented. It is also retrospective data, which means the findings cannot be interpreted as psychopathy being a prospective predictor of gun violence. However, this multi-source data collection is a strength to the study. Using self-report for psychopathy and official records for violent crime prevents common method variance if data were collected from the same source (e.g., self-report). Nevertheless, future studies would benefit from integrating multiple data sources to corroborate findings. Lastly, it was not possible to create a comparison violent crime group that used another type of weapon (e.g., a knife) because this data was not collected. This line of inquiry would be important to test if the present findings were related to gun violence or just weapon-related violence.

In summary, the present study demonstrates that women who engage in gun violence are characteristically different from violent and non-violent women. This should encourage future research to tease apart known risk factors for violence to understand if they replicate or differ from gun violence. Further, as these studies roll out, it is strongly encouraged that existing interventions tailor their strategies to address these firearm specific risk factors. It is also encouraged that new interventions be developed to help target the growing public health crisis of gun violence.

## Data Availability Statement

The datasets presented in this article are not readily available because in accordance to prison administrators request, the informed consent stated that the data would not be shared beyond the immediate study team. Requests to access the datasets should be directed to corresponding author.

## Ethics Statement

The studies involving human participants were reviewed and approved by the University of Hawai’i IRB. The patients/participants provided their written informed consent to participate in this study.

## Author Contributions

NT conducted the data collection and analyses and wrote of the manuscript.

## Conflict of Interest

The author declares that the research was conducted in the absence of any commercial or financial relationships that could be construed as a potential conflict of interest.

## Publisher’s Note

All claims expressed in this article are solely those of the authors and do not necessarily represent those of their affiliated organizations, or those of the publisher, the editors and the reviewers. Any product that may be evaluated in this article, or claim that may be made by its manufacturer, is not guaranteed or endorsed by the publisher.
